# Characterization of Neonatal Vocal and Motor Repertoire of Reelin Mutant Mice

**DOI:** 10.1371/journal.pone.0064407

**Published:** 2013-05-21

**Authors:** Emilia Romano, Caterina Michetti, Angela Caruso, Giovanni Laviola, Maria Luisa Scattoni

**Affiliations:** 1 Behavioural Neuroscience Section, Department of Cell Biology & Neuroscience, Istituto Superiore di Sanità, Rome, Italy; 2 Neurotoxicology and Neuroendocrinology Section, Department of Cell Biology & Neuroscience, Istituto Superiore di Sanità, Rome, Italy; 3 Bambino Gesù Children's Hospital Istituto Di Ricovero e Cura a Carattere Scientifico, Rome, Italy; Rutgers University, United States of America

## Abstract

Reelin is a large secreted extracellular matrix glycoprotein playing an important role in early neurodevelopment. Several genetic studies found an association between RELN gene and increased risk of autism suggesting that reelin deficiency may be a vulnerability factor in its etiology. Moreover, a reduced reelin expression has been observed in several brain regions of subjects with Autism Spectrum Disorders. Since a number of reports have documented presence of vocal and neuromotor abnormalities in patients with autism and suggested that these dysfunctions predate the onset of the syndrome, we performed a fine-grain characterization of the neonatal vocal and motor repertoire in reelin mutant mice to explore the developmental precursors of the disorder. Our findings evidence a general delay in motor and vocal development in heterozygous (50% reduced reelin) and reeler (lacking reelin gene) mutant mice. As a whole, an increased number of calls characterized heterozygous pup's emission. Furthermore, the typical ontogenetic peak in the number of calls characterizing wild-type pups on postnatal day 4 appeared slightly delayed in heterozygous pups (to day 6) and was quite absent in reeler littermates, which exhibited a flat profile during development. We also detected a preferential use of a specific call category (*two-components*) by heterozygous and reeler mice at postnatal days 6 and 8 as compared to their wild-type littermates. With regard to the analysis of spontaneous movements, a differential profile emerged early in development among the three genotypes. While only slight coordination difficulties are exhibited by heterozygous pups, all indices of motor development appear delayed in reeler mice. Overall, our results evidence a genotype-dependent deviation in ultrasonic vocal repertoire and a general delay in motor development in reelin mutant pups.

## Introduction

A number of studies implicate the glycoprotein reelin in the etiology of several neurodevelopmental disorders such as schizophrenia [1], [2], lissencephaly [3] and autism [4]–[6]. Reelin is a protein of the extracellular matrix involved in regulation of embryonic brain development [7], [8]. In particular, this protein plays an important role in regulating neuronal migration and brain lamination and promoting dendrite maturation, axonal growth, and the establishment of synaptic contacts [5], [9]–[12]. In addition, reelin seems to act after embryonic development in synaptogenesis and synaptic plasticity [13].

Spontaneous reeler mutant mouse (Rl, lacking Reelin gene) presents lamination defects in cerebral cortex, hippocampus and cerebellum. The cerebellum shows a decreased number of Purkinje cells [14] leading to a severe cerebellar hypoplasia and an ataxic phenotype characterized by uncoordinated and unsteady gait, imbalance, tremors and usually early death around the time of weaning [15]–[19]. Due to its serious physical impairments, reeler mice are not considered as a reliable animal model for basic behavioral research and its use has been so far limited to the study of neuronal migration and of the etiology of human lissencephaly [3], [20].

Unlike Rl mice, levels of reelin in heterozygous mice (Het) are reduced by 50% compared to wildtype (Wt) and the lamination defects in the CNS are absent. This molecular depletion produces a range of subtle neurobehavioral consequences including increased impulsivity and disinhibited behavior [6], [21], [22], impaired executive function [23], [24], associative learning deficit [25], reduced social motivation [26], [27] and reduced pre-pulse inhibition [28].

Reduced reelin expression has been observed in several brain regions of subjects with autism [4], [29], [30]. Post-mortem studies [29], showed that reelin is reduced in cerebellum (∼40%), superior frontal (∼70%) and parietal (∼70%) cortices. These brain regions have been implicated in the mediation of the three core behaviors that are impaired in autism: social behavior, language and communication, and repetitive and stereotyped behaviors. In particular, the frontal and parietal cortices influence the planning and organization of behaviour and are closely related to the recognition of language and memory for words. The cerebellum has been proposed to serve as an integrative area providing ‘correct predictions about the relationship between sensory stimuli’ and its dysfunctions may directly relate to cognitive impairments.

Moreover, several genetic studies found an association between RELN gene and increased risk of autism [31]–[36]. Altogether these genetic and molecular studies suggest that the reelin deficiency may be a vulnerability factor in the pathology of autism. Since several reports have documented the presence of vocal and neuromotor abnormalities in patients with autism and suggested that these dysfunctions predate the onset of the syndrome, we performed a fine-grain characterization of the neonatal vocal and motor repertoire in reelin mutant mice to explore the developmental precursors of this disorder [37]. The elucidation of the developmental onset of autism will be crucial in designing early intervention strategies to reduce the incidence and impact of autism-related abnormalities.

To address the hypothesis that Het and Rl mice display motor and vocal alterations [38] and that autism-like phenotypes [39] in these mutant mice can be detected at an early developmental stage, we analyzed development of ultrasonic vocalization (USV) patterns and spontaneous motor behavior throughout the first two postnatal weeks. Ultrasonic vocalizations, emitted by mouse pups in response to separation from the lactating mother, are considered a reliable index of social motivation and provide a very sensitive insight into the early emotional development [40]–[43] thus representing a suitable and reliable tool for the identification of the early communication deficits in autism animal models [21], [37], [38], [44]–[50]. The primary goal of the present study was to detect any unusual component of vocalizations in Rl and Het mice at infant stages, relevant to the absence of crying, and the unusual guttural grunts and squeals, reported for some babies that were later diagnosed with autism [51], [52].

Our results evidence a genotype-dependent deviation in ultrasonic vocal repertoire and a general delay in motor development in reelin mutant pups.

## Materials and Methods

### Animals

B6C3Fe heterozygous female and male mice, originally purchased from Jackson Laboratories (USA) were bred in our laboratory. Two females were housed with one male in (33 cm×13 cm×14 cm) Plexiglas boxes, with sawdust bedding and a metal top. After two weeks of mating, male mice were removed, dams were housed individually in Plexiglass cages (33×13×14 cm), and daily checked for delivery. Mice were maintained on a reversed 12∶12 h light∶ dark cycle (lights on at 18∶30 h). The temperature was maintained at 21±1°C, relative humidity (60±10%). Mice genotype was determined at weaning (pnd 25) by RT-PCR on tail samples. Mice are weaned into cages of same sex pairs. After weaning mice were housed in pairs within Plexiglass cages (27×21×14 cm), with sawdust bedding. Animals were provided drinking water and a complete pellet diet (Mucedola, Settimo Milanese, Italy) ad libitum. Pups were tattooed on the paw with animal tattoo ink (Ketchum permanent Tattoo Inks green paste, Ketchum Manufacturing Inc., Brockville ON Canada) by loading the ink into a 30G hypodermic needle and inserting the ink subcutaneously through the needle tip into the center of the paw. The procedure was performed at two days of age, immediately after behavioral testing. The procedure causes only minor brief pain and distress and does not require the use of anesthesia. All procedures were in accordance with the European Communities Council Directive (86/609/EEC) and formally approved by Italian Ministry of Health.

### Ultrasonic vocalizations in separated pups

Litters chosen for testing contained more than six pups. Body weights and body temperatures of pups were measured after the ultrasonic vocalization test on pnd 2, 4, 6, 8 and 12. On each day of testing, each pup was placed into an empty glass container (diameter, 5 cm; height 10 cm), located inside a sound-attenuating styrofoam box, and assessed for ultrasonic vocalizations during a three minute test. At the end of the three minute recording session, each pup was weighed and its axillary temperature measured by gentle insertion of the thermal probe in the skin pocket between upper foreleg and chest of the animal for about 30 seconds (Microprobe digital thermometer with mouse probe, Stoelting Co., Illinois, USA). No differences in patterns of calling were detected in a comparison of male and female pups, therefore data were collapsed across sex.

An Ultrasound Microphone (Avisoft UltraSoundGate condenser microphone capsule CM16, Avisoft Bioacoustics, Berlin, Germany) sensitive to frequencies of 10–180 kHz, recorded the pup vocalizations in the sound-attenuating chamber. The microphone was placed through a hole in the middle of the cover of the styrofoam sound-attenuating box, about 20 cm above the pup in its glasses container. The temperature of the room was maintained at 22°C. Vocalizations were recorded using Avisoft Recorder software (Version 3.2). Settings included sampling rate at 250 kHz; format 16 bit. For acoustical analysis, recordings were transferred to Avisoft SASLab Pro (Version 4.40) and a fast Fourier transformation (FFT) was conducted. Spectrograms were generated with an FFT-length of 1024 points and a time window overlap of 75% (100% Frame, Hamming window). The spectrogram was produced at a frequency resolution of 488 Hz and a time resolution of 1 ms. A lower cut-off frequency of 15 kHz was used to reduce background noise outside the relevant frequency band to 0 dB. Call detection was provided by an automatic threshold-based algorithm and a hold-time mechanism (hold time: 0.01 s). An experienced user checked the accuracy of call detection, and obtained a 100% concordance between automated and observational detection. Parameters analyzed for each test day included number of calls, duration of calls, total calling time, qualitative and quantitative analyses of sound frequencies measured in terms of frequency and amplitude at the maximum of the spectrum.

### Qualitative analyses

We classified every USVs emitted at pnd 2, 4, 6, 8, 12, in nine distinct categories, based on internal pitch changes, lengths and shapes, using our previously published categorization [37]. Classification of USVs included nine waveform patterns described below: 1) *Complex* calls displayed one component containing two or more directional changes in pitch, each ≥6.25 kHz; 2) *Two-component* calls consisted of two components: a main call (flat or downward) with an additional punctuated component towards the end; 3) *Upward*-modulated calls exhibited a continuous increase in pitch that was ≥12.5 kHz, with a terminal dominant frequency at least 6.25 kHz more than the pitch at the beginning of the vocalization; 4) *Downward*-modulated calls exhibited a continuous decrease in pitch that was ≥12.5 kHz, with a terminal dominant frequency at least 6.25 kHz less than the pitch at the beginning of the vocalization; 5) *Chevron* calls resembled an ‘inverted-U’, which was identified by a continuous increase in pitch ≥12.5 kHz followed by a decrease that was ≥6.25 kHz; 6) *Short* calls were punctuated and shorter than 5 ms; 7) *Composite* calls were formed by two harmonically independent components, emitted simultaneously; 8) *Frequency steps* were instantaneous frequency changes appearing as a vertically discontinuous “step” on a spectrogram, but with no interruption in time; 9) *Flat* calls displayed a constant beginning and the ending of the pitch frequency remained constant (≤3 kHz of each other).

Call category data were subjected to two different analyses: a) genotype dependent effects on the frequency of vocalizations emitted by each subject at pnd 2, 4, 6, 8, 12; b) genotype-dependent effects on the probability of producing calls from each of the nine categories of USV, as described below under Statistical analysis.

### Righting reflex

After each USV recording session, each pup was placed on its back over a flat surface, and the time needed to return to the natural position (all four paws on the floor) was measured. This reflex requires complex coordination between head, trunk, and paws. The reflex was tested once with a cut-off latency of 60 s. Latencies were measured in seconds, using a stopwatch for righting reflex.

### Spontaneous movements

Concomitant with the vocalizations recording on pnd 2, 4, 6, 8 and 12, mouse pups were also videorecorded for analysis of spontaneous movements. Frequency and duration of behavioral items were analyzed by an observer blind to mouse genotype. We used the NOLDUS OBSERVER software V 10XT (Noldus Information Technology, Wageningen, NL, USA) to score the videotapes. In accordance with previous studies focused on neonatal rodent behavior [53]–[56], the following behavioral patterns were scored: locomotion (general translocation of the body of at least 1 cm in the glass container), immobility (no visible movement of the animal when placed with all the four paws on the floor), side (no visible movement of the animal when laying on the side), head rising (a single rising of the head up and forward), head shaking (a single lateral displacement of the head), face washing (forepaws moving back and forth from the ears to the snout and mouth), wall climbing (alternating forelimb placing movements on the wall of the container), nose probing (pushing the snout against floor or walls of the apparatus), pivoting (locomotor activity involving the front legs alone and resulting in laterally directed movements), circling (circular locomotor activity involving the all legs and resulting in laterally directed movements), and curling (roll, vigorous side-to-side rolling movements while on the back; curl, a convex arching of back while on side or back, bringing head in a closer opposition to hump/hindlimb region).

### Statistical analysis

A mixed-model Analysis of Variance (ANOVA) with Repeated Measures was performed to analyze genotype-dependent effects on neonatal USVs and spontaneous movement responses, with the genotype (Wt vs Het vs Rl) as factor and pnd or call categories as the repeated measures.Probability of vocalizations within strain was calculated as number of calls in each category for each subject/total number of calls analyzed in each subject and standardized by angular transformation. Since no sex differences were detected, data were collapsed across sex. Post-hoc comparisons were performed using Tukey HSD Test only when a significant F-value was determined. For all comparisons, significance was set at P = 0.05.

## Results

### Body weight gain

Body weight did not differ between genotypes [genotype: F(2,73) = 1.00, ns; genotype x day: F(8,292) = 1.603, ns] and, as expected, progressively increased with age [F(4,292) = 232.12; p<0.0001)] (data not shown).

### Pup separation vocalizations

**Figure 1:**
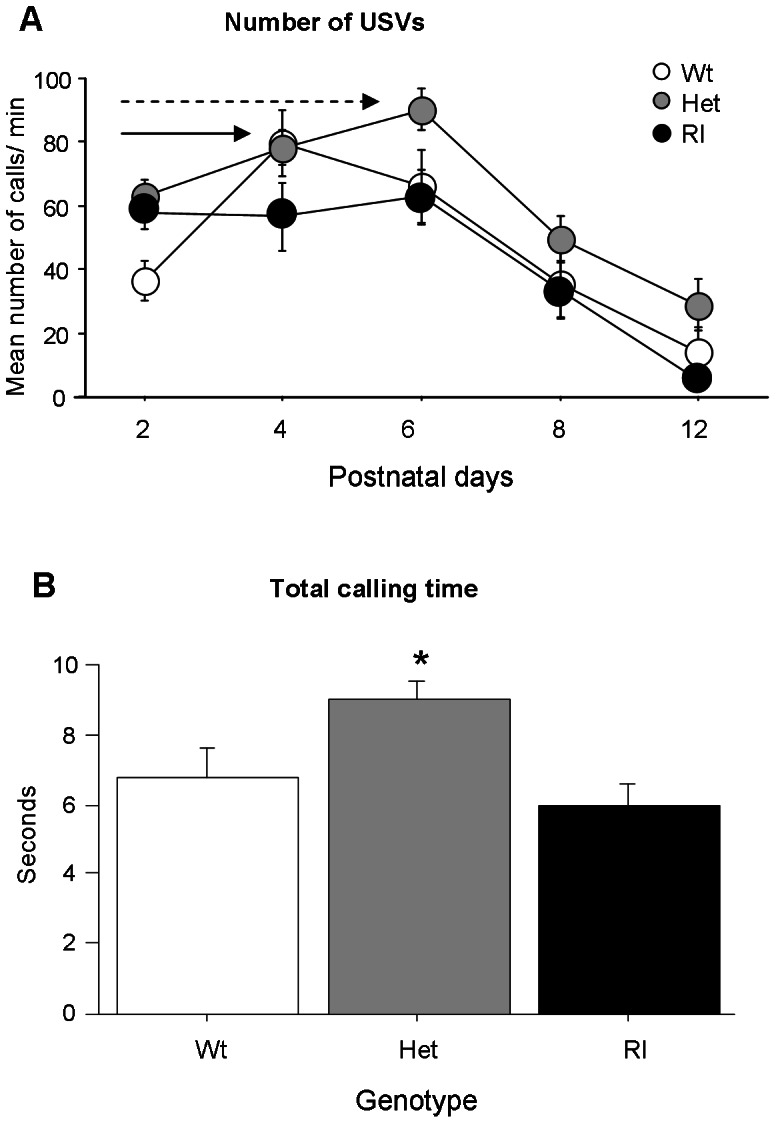
Ultrasonic vocalizations (USVs) in Wt, Het and Rl pups. A) Number of vocalizations on postnatal day (pnd) 2, 4, 6, 8 and 12 in response to social separation during a three minute session. Data are expressed as mean number of calls/min ± SEM. The arrows (uninterrupted for Wt, dashed for Het) indicate the peak of emission of USVs. B) Total calling time: number of vocalizations x mean duration. The graph shows genotype effect on total calling time (data pooled from all ages considered). N = 17 Wt, 43 Het, 16 Rl pups.

Changes of ultrasonic vocalizations (USVs) over time were characterized by an inverted U-shape across the first 12 postnatal days of age, as confirmed by the main effect of day [day: (F (4,292) = 26.412, p<0.0001)] (Figure 1A). Wildtype pups showed a peak of emission at pnd 4, while it was slightly delayed to pnd 6 in Het pups. By contrast, the profile of emission in Rl mice appeared quite flat, without a peak, across the five days of testing. Wildtype and Het pups emitted same number of calls at pnd 4. Total number of vocalizations varied across genotypes [F(2,73) = 5.601, p<0.005)]. In particular, Het emitted a significantly higher number of calls than Wt and Rl mice. No differences in genotype were found for mean duration, peak frequency and peak amplitude of USVs [duration: (F(2,73) = 2.575, ns; peak frequency: (F(2,73) = 0.459, ns; peak amplitude: F(2,73) = 0.041, ns]. As shown in Figure 1B, Het pups emitted USVs for longer time than the other genotypes [F(2,73) = 5.042, p<0.05]. (Figure 1B).

No differences were detected in body temperatures of Rl, Het and Wt as measured after each separation test [genotype: F(2,73) = 0.269, ns; genotype x day: F(8,292) = 0.790, ns], (data not shown).

### Classification of ultrasonic vocalizations into distinct categories

**Figure 2:**
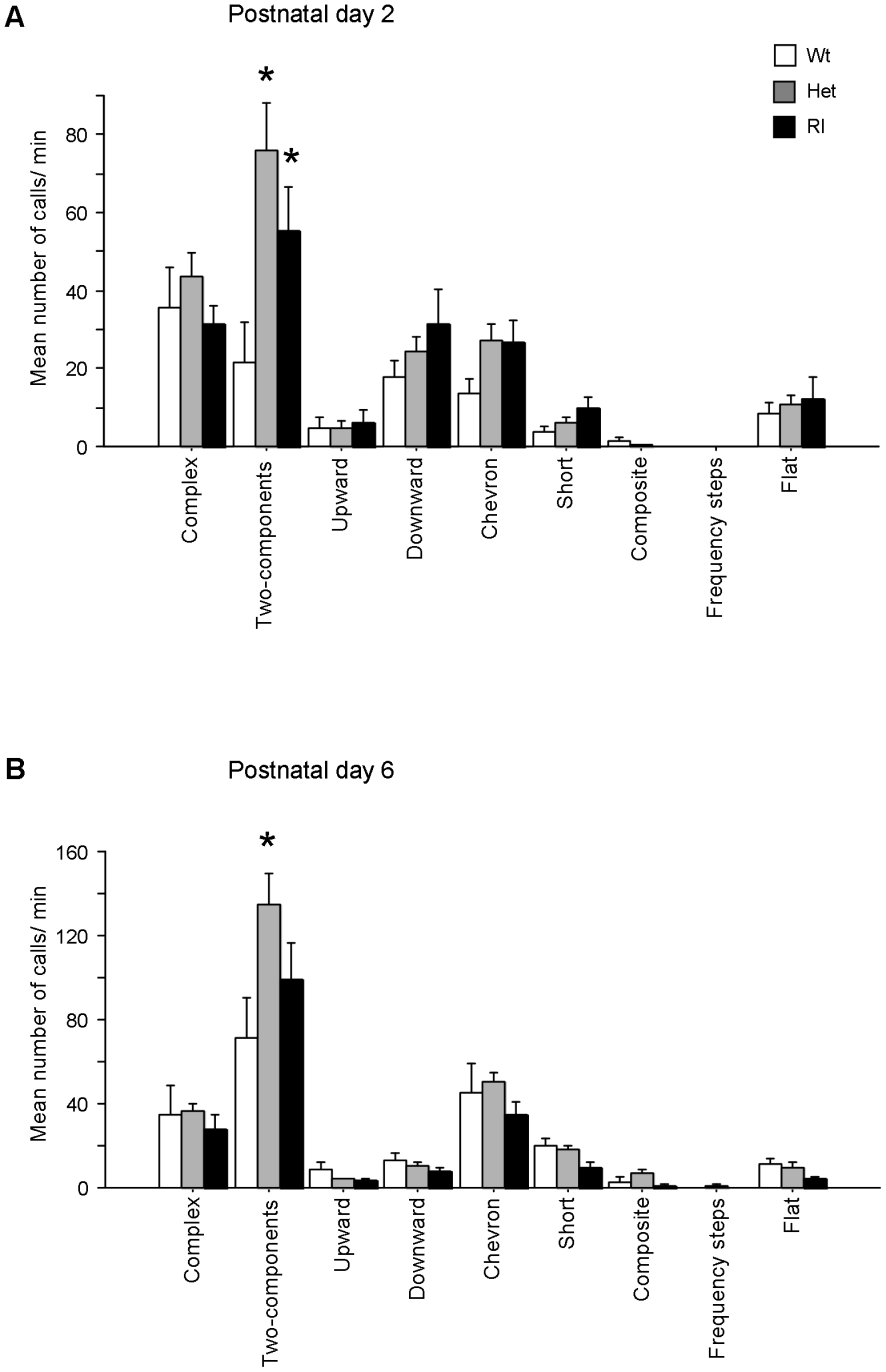
Production of ultrasonic vocalizations by call category. A) Frequency of ultrasonic vocalizations at pnd 2 (post hoc p<0.05 for Het and Rl *vs* Wt). B) Frequency of ultrasonic vocalizations at pnd 6 (post hoc p<0.05 for Het *vs* Wt and Rl).

Figure 2A illustrates genotype-dependent variation on frequency of calls at pnd 2 [F (2,73) = 5.55, p<0.005)]. Het and Rl pups emitted a significantly higher number of *two-component* calls than Wt pups [p<0.05 after post hoc comparison performed on genotype x calls subtype interaction: F (16,584) = 2.39, p<0.005]. As illustrated in Figure 2B, a genotype-dependent effect have been found also at pnd 6 [genotype: (F (2,73) = 2.96, p = <0.05)], with Het emitting a significantly higher number of *two-component* calls than the other genotypes [genotype x calls subtype interaction: F (16,584) = 2.57, p<0.0001]. No differences among genotypes were evidenced on other subtypes of calls later on in development (see Figure S3).

### Pattern of sonographic structure among genotypes

**Figure 3:**
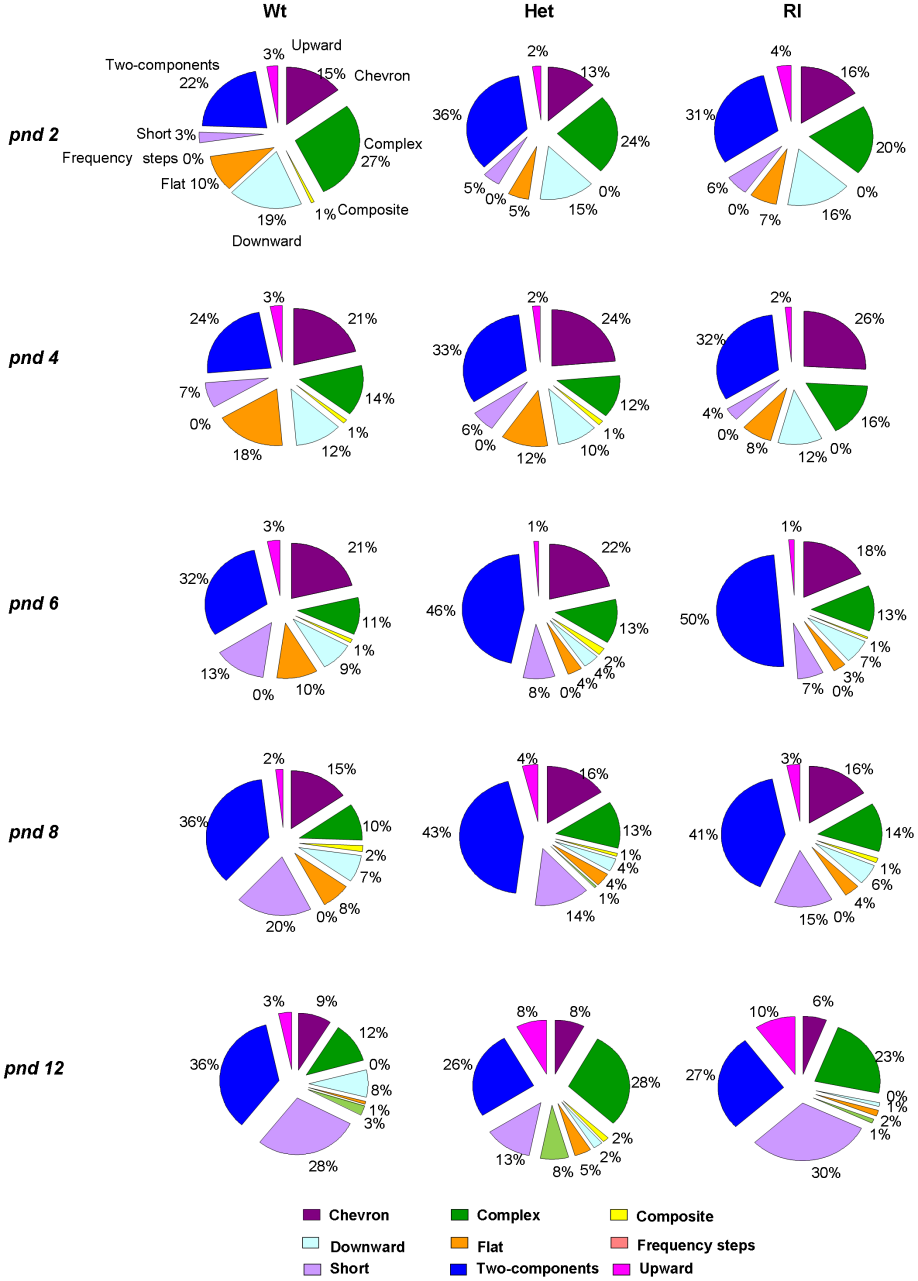
Pie graphs show the percentages of the different call categories for the three genotypes, Wt, Het, Rl during five days of testing (pnd 2, 4, 6, 8, 12). Percentages were calculated in each genotype as number of calls in each category for each subject/total number of calls analyzed for each subject. Number of total calls analyzed at pnd 2: Wt = 1823; Het = 8301; Rl = 2776. Pnd 4: Wt = 4064; Het = 10015; Rl = 2708. Pnd 6: Wt = 3522; Het = 11642; Rl = 3017. Pnd 8: Wt = 2511; Het = 6613; Rl = 1603. Pnd 12: Wt = 705; Het = 3692; Rl = 614.

**Figure 4:**
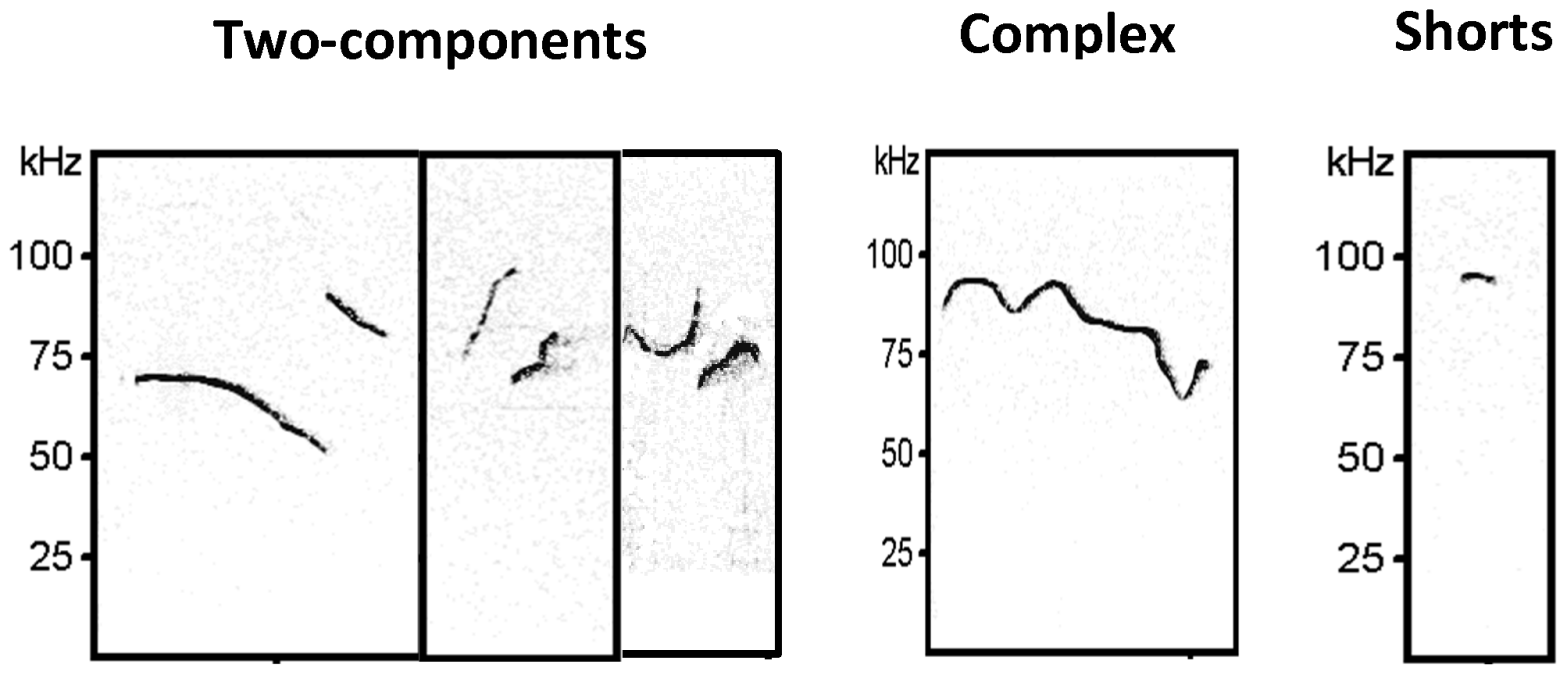
Exemplary sonograms of two-component, complex and short calls.

Proportions of calls within each category are shown in Figure 3. Wt, Het and Rl pups emitted a wide spectrum of call categories. At pnd 2, Wt pups mainly emitted *chevron*, *complex*, *downward*, *flat* and *two-component* calls, along with low prevalence in production of *upward*, *composite*, *frequency steps* and *short* calls. On pnd 4, Wt pups showed a decrease in the emission of the *complex* calls in favor of the *flat* calls. On pnd 6, as well as on pnd 8, a reduction of the *flat* and *downward* calls appeared in favor of *two-component* and *short* calls. This profile appeared even more pronounced on pnd 12, with general vocal repertoire reduced and mainly defined by the concomitant presence of two types of calls: *two-components* and *shorts*. Het and Rl pups (see second and third column), already on pnd 2, exhibited a differential vocal repertoire in comparison to Wt controls. Indeed, mice of both mutant genotypes emitted a high proportion of *two-component* calls and a low proportion of *flat* calls, whereas other types of vocalizations remain unchanged. This profile was still there on pnd 4. The data on pnd 6 indicate that the vocal repertoire of Het and Rl pups was represented by 50% of *two-component* calls. This profile was still present on pnd 8, then reducing later on. In fact, on pnd 12, Het pups still persisted in emitting primarily the *complex* and *two-component* calls, while Wt emission was characterized by a prevalence of *two-component* and *short* calls. At this age, Rl pups showed a vocal repertoire characterized by the *two-component*, *short* and *complex* calls, indicative of a phenotype halfway between Wt and Het pups (see exemplary sonograms in Figure 4).

Proportions of calls within genotype and sex are shown in Figures S1 and S2.

**Figure 5:**
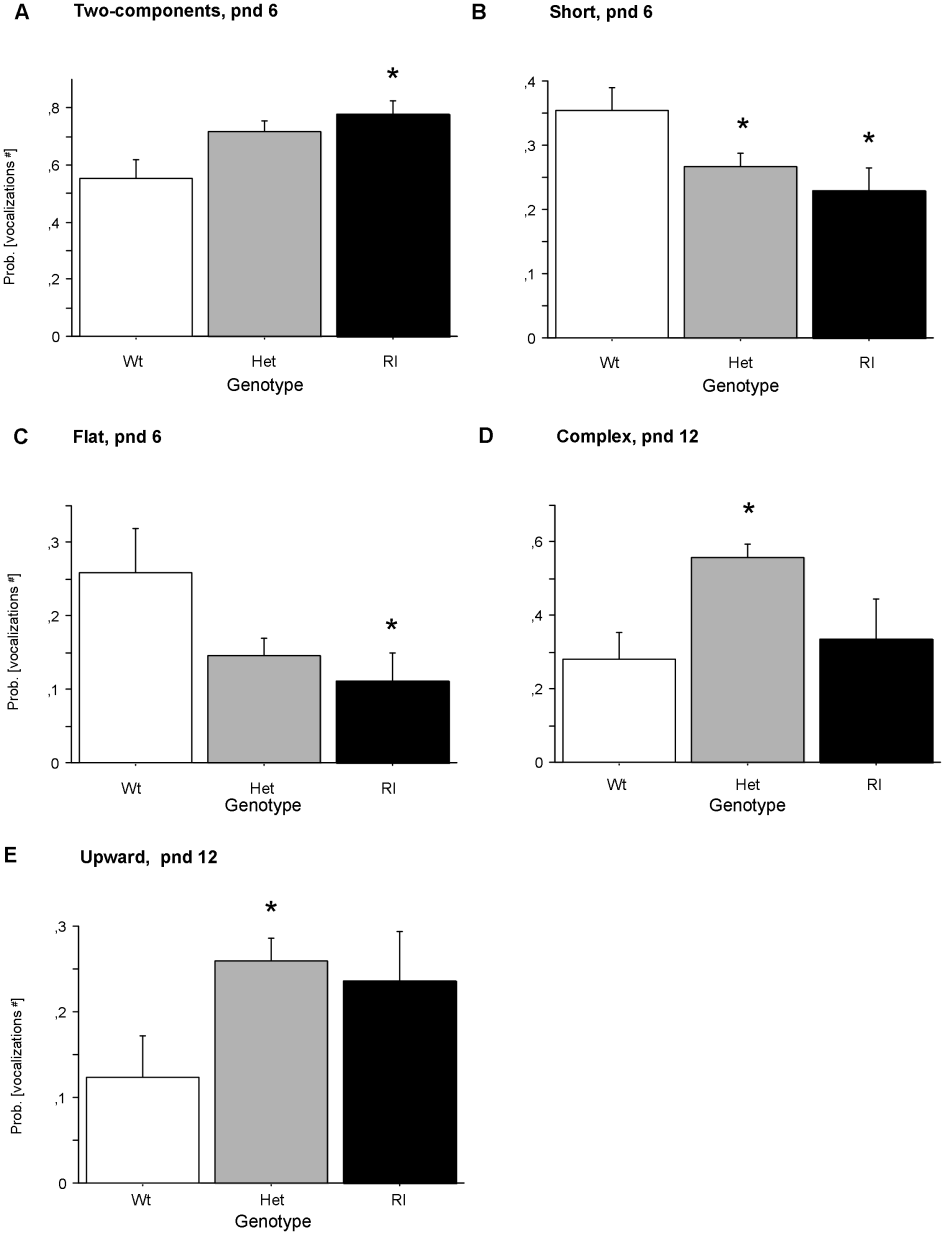
Production of calls within genotype. Probability of producing calls from each of the nine categories of USV. ^#^Data were expressed by angular transformation. A) *Two-component* calls at pnd 6, B) *Short* calls at pnd 6, C) *Flat* calls at pnd 6, D) *Complex* calls at pnd 12, and E) *Upward* calls at pnd 12. *p<0.05.

When analyzing each USV category separately on different postnatal days (see Figure 5, Panels A, B, C, D, E), a main effect of genotype was found for the probability of producing different categories of calls (this index is obtained by calculating the number of calls in each category for each subject/total number of calls analyzed for each subject): [*two-components*, pnd 6: F(2,70) = 3.692, p<0.05; *short*, pnd 6: F(2,70) = 3.328, p<0,05; *flat*, pnd 6: F(2,70) = 3.573, p<0,05; *upward*, pnd 12: F(2,35) = 3.367, p<0.05; *complex*, pnd 12: F(2,35) = 7.845, p<0.001]. Specifically, on pnd 6, Wt pups emitted less *two-component calls* than Rl pups, more *short* calls than Het and Rl pups, and more *flat* calls than Rl pups (post hoc, p<0.05). At pnd 12, Het pups emitted more *complex* and *upward* calls than Wt subjects (post hoc, p<0.05).

### Righting reflex

**Figure 6:**
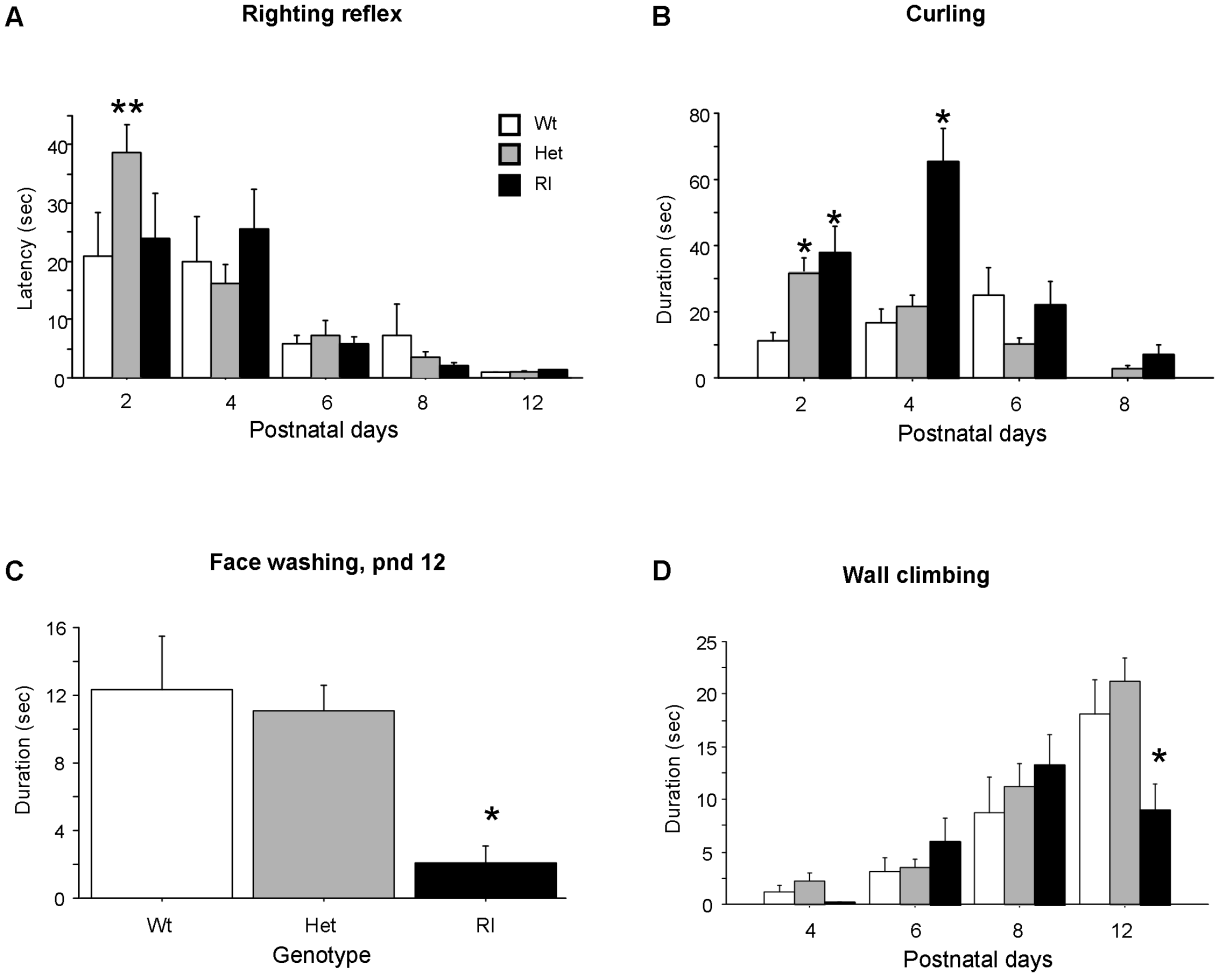
Latency and/or duration of behavioral patterns shown by Wt, Het and Rl pups on pnd 2, 4, 6, 8 and 12 during a 3-min session. See methods for full description of neonatal behaviors. A) Righting reflex latencies. Pups acquired the righting reflex response at different rates, with Wt and Rl showing shorter latencies than Het at pnd 2. B) Curling, C) Face washing and D) Wall climbing. Data are expressed as mean ± SEM. ** p<0.01 and *p<0.05; N = 17 Wt, 43 Het, 16 Rl pups.

The righting reflex, measured as latency to turn back onto all four paws when placed on the back [57], was tested at pnd 2 to 12 after the USV and spontaneous movements recording session. As expected, *righting reflex* latencies differed significantly across pnd [F(4,204) = 24.290, p<0.0001], with all genotypes reaching the full development of the reflex on pnd 12. Post hoc comparisons performed on the two way interaction genotype x day [F(8,204) = 2.241, p<0.05] indicated that Het spent more time to turn their body than Wt and Rl pups on pnd 2 (p<0.01) (see Figure 6A).

### Analysis of spontaneous movements

*Curling* behavior is measured by time spent from pups rolling on the back. This behavior is associated to the development of the *righting reflex*. *Curling* duration rapidly decreased across the five days of observation and disappeared on pnd 12 [main effect of age: F(3,219) = 26.238; p<0.0001]. A main effect of genotype was found [F(2,73) = 11,151; p<0.0001]. Post hoc comparisons performed on the interaction genotype x days of testing [F(6,219) = 8.149; p<0.0001] showed that *curling* durations in Het and Rl mice were longer than Wt on pnd 2 (p<0.05), and persisted longer in Rl mice on pnd 4 (p<0.05), thus suggesting a motor coordination deficit in Het and Rl pups in getting an upright position (see Figure 6B).

*Face washing* requires coordination and equilibrium since the pup is standing up on its hindlimb and washing its face with forelimbs. For this reason, it was exhibited and analyzed only at day 12 of observation. A main effect of genotype was found for frequency and duration of *face washing* [frequency: F(2,73) = 4.726; p<0.05; duration: F(2,73) = 5.822; p<0.005], with Rl pups being significantly much less involved than Wt and Het subjects, (see Figure 6C).

A similar profile was observed for frequency and duration of *wall climbing*, as confirmed by a significant genotype x day interaction [F(6,219) = 2,899; p<0.05; F(6,219) = 2,692; p<0.05, respectively], (see Figure 6D). Post hoc comparisons showed that, on pnd 12, Rl pups spent consistently less time climbing the walls of the glass container than Wt and Het pups (P<0.05).

**Figure 7:**
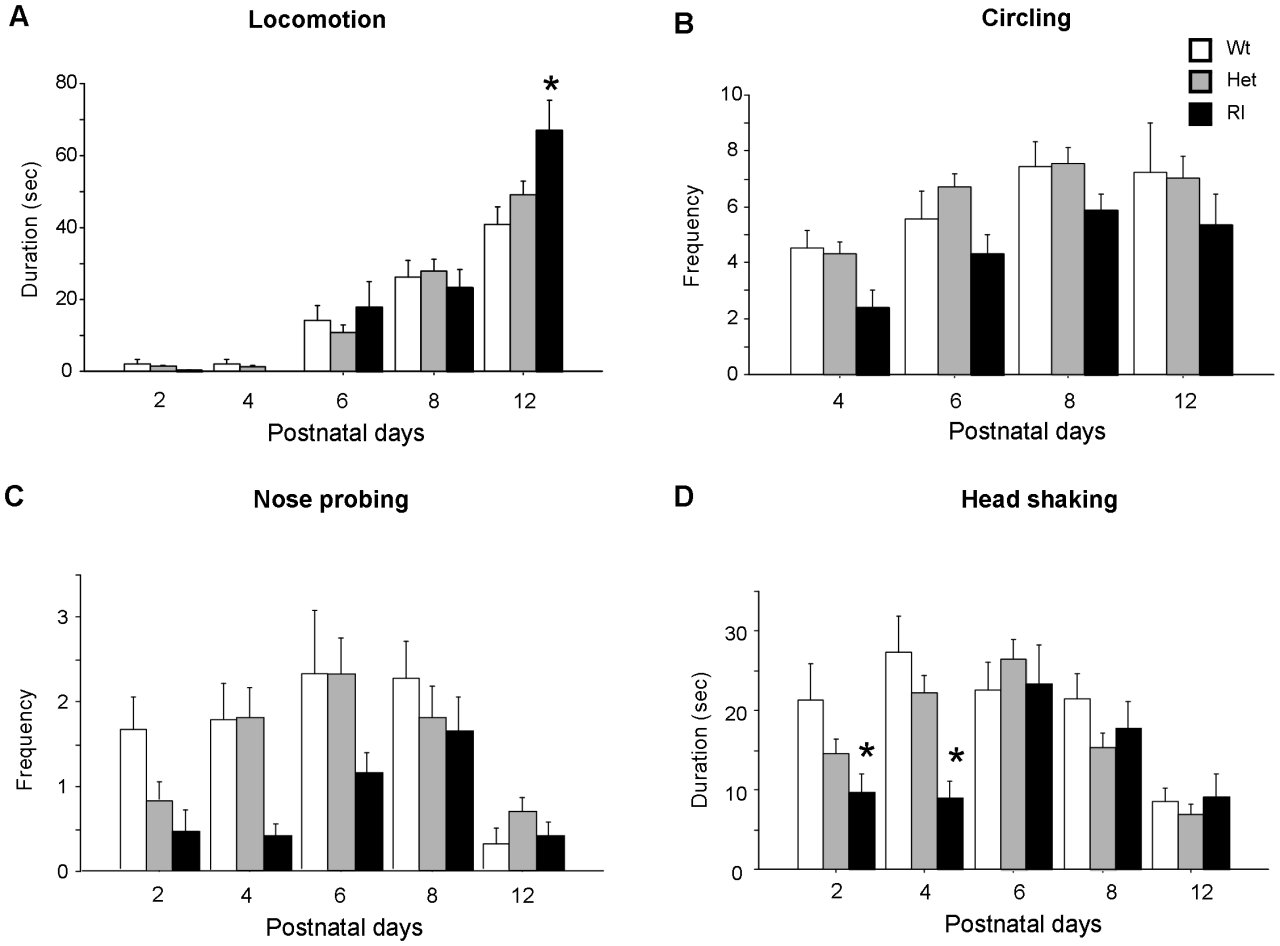
Frequency and/or duration of behavioral patterns shown by Wt, Het and Rl pups on pnd 2, 4, 6, 8 and 12 during a 3-min session. A) Locomotion, B) Circling, C) Nose probing, D) Head shaking. Data are expressed as mean ± SEM. *p<0.05; N = 17 Wt, 43 Het, 16 Rl pups.

Time spent in *locomotion* rapidly increased across the five days of observation [F(4,292) = 102.346; p<0.0001]. Post hoc comparisons performed on the interaction genotype x time intervals [F(8,292) = 2.662; p<0.05] indicated that Rl mice were more active than Wt and Het mice on pnd 12 (P<0.05), (see Figure 7A).

Analysis of frequency of *circling* behavior yielded a significant main effect of genotype [F(2,73) = 3.972; p<0.05)], with Rl mice exhibiting a lower number of circling episodes than the other genotypes at all days of testing (see Figure 7B). A similar profile was evident for duration of circling but the genotype effect just missed the statistical significance [F(2,73) = 2.393; P = 0.098].

ANOVA performed on frequency of nose probing detected a main effect of genotype [F(2,73) = 4.017; p<0.05], with Rl mice performing less nose probing than Wt and Het pups (see Figure 7C). In general, the profile of frequency of nose probing exhibited by Wt and Het pups was characterized by an inverted U-shape profile across the five days of observation, with a peak on pnd 6 [time intervals: (F(4,292) = 7.526, p<0.0001]. Instead, the nose probing profile of Rl pups appeared delayed in time and showed its peak on pnd 8 (see Figure 7C).

A main effect of age was found for *head shaking* [frequency: F(4,292) = 14.551; p<0.0001; duration: F(4,292) = 7.888; p<0.0001]. Post hoc comparisons performed on the genotype x time intervals interaction [frequency: F(8,292) = 2.767; P = 0.005; duration: F(2,73) = 2.941; p<0.05] revealed that Rl pups spent significantly less time in shaking their heads than Wt on pnd 2 (p<0.05) and less time than Wt and Het littermates on pnd 4 (p<0.05), (see Figure 7D).

No differences between genotypes were observed for *head rising*, *side*, *pivoting*, *and immobility* behaviors (data not shown).

## Discussion

Reelin is a glycoprotein playing a role in regulating neuronal migration and brain lamination during development. The reduced or complete lack of reelin signaling impairs neuronal connectivity and synaptic plasticity leading ultimately to the cognitive deficits present in autism and schizophrenia. So far, limited studies study have investigated the contribution of reelin deficiency to the establishment of the early motor and social/communicative deficits present in these neurodevelopmental disorders [6], [38]. To this aim, the present study provides, for the first time, a fine-grain characterization of neonatal vocal and motor repertoires in reelin mutant mice, a genetic line widely used as animal model of autism and schizophrenia. In rodent models of neurodevelopmental disorders, it is critical to conduct behavioral phenotyping during the early developmental period in order to document the precise onset of symptoms, identify transient signs, and provide a basis for the timing of early intervention [58], [59]. Moreover, performing experiments during the first two postnatal weeks allowed us to include in the study also the homozygous reeler mouse, presenting an impaired phenotype generally leading to death shortly after weaning. The inclusion of Rl mice is crucial for the assessment of dose-dependent effects in genetic vulnerability [6].

Heterozygous reeler pups separated from their mothers and siblings at pnd 2, 4, 6, 8 and 12 emitted significantly more calls and for longer time than their wildtype and homozygous littermates. Moreover, a different profile of emission was detected in Het and Rl mice in comparison to wildtype pups. In fact, while wildtype pups showed a peak of emission at pnd 4, Het's peak of emission was slightly postponed to pnd 6, and profile of emission in Rl mice appeared quite flat, without a peak, indicative of a potential delay in the emotional/communicative development in this mutant line. No differences were detected in body temperature upon USVs recording excluding the possibility of unusual thermoregulation in reeler mice. An increased number of USVs has been detected also in other animal models of autism such as BTBR T^+^ tf/J, Shank2 −/− and Mecp2 null mice, Tsc1 +/− and Tsc1−/− conditional knockouts and mice with a chromosome 15q11–13 maternal deletion or a paternal duplication [37], [46], [60]–[64].

Our USV data are partially in contrast with previous data collected in this model [38] showing a reduced number of USV in Het and Rl pups as compared to wildtype pups at pnd 7. Important differences in the experimental procedures, such as room temperature, may be implicated. In fact, the temperature of our experimental room was maintained at 22°C, while in Laviola's study [38] the temperature was set at 26°C. Body temperature strongly affects ultrasonic vocalization emission, with higher temperature reducing the number of vocalizations [65]. Moreover, Laviola and colleagues measured ultrasonic vocalizations by a bat-detector tuned between 40 kHz and 60 kHz and were able to detect only calls within this range. Indeed, in our technologically improved and updated experimental setting, we have been able to detect any vocalization emitted in the frequency range of 10–180 kHz resulting in an increasing number of vocalizations recorded in each genotype.

We further investigated the specific types of calls emitted by the three genotypes at all days of testing. Specifically, according to our previous paper on pup vocalizations [37], we classified waveform patterns into nine categories of calls designated as *complex*, *composite*, *downward*, *flat*, *frequency steps*, *short*, *two-components*, *upward* and *chevron*. To our knowledge, this is the first report about a detailed analysis of pup vocal repertoire throughout the first two postnatal weeks of age. Aim of this deeper investigation was to detect subtle alterations in normative developmental aspects that could be potentially transferred to human studies for early detection of ASD [66]. Analysis of the vocal repertoire revealed a significant difference between genotypes in the use of specific categories. Wildtype pups, at pnd 2, emitted mainly 5 out of 9 categories (*chevron*, *complex*, *downward*, *flat* and *two-components*) while at pnd 12 their vocal repertoire appeared more limited (64%) and narrowed to two categories of vocalizations, such as *two-component* and *short* calls, and to a low number (29%) of *chevron*, *complex* and *downward* calls. This analysis allowed us to evaluate the development of the vocal profile of wildtype mice from a neonatal to a more adult-like repertoire. In fact, the call distributions showed by wildtype pups at pnd 12 are similar to what B6 males and females emitted in adult social contexts [67]. Het and Rl pups emitted a restricted repertoire of calls at pnd 6 and 8 in comparison to Wt pups, limiting their vocal repertoire to the *two-component* (about 50%), *chevron* (about 20%) and *complex* calls (13%). At pnd 12, Het pups still persisted in emitting primarily the *complex* and *two-component* calls, while Rl pups showed a vocal repertoire characterized by the *two-component*, *short* and *complex* calls, indicative of a phenotype halfway between Wt and Het pups. As a whole, from a detailed analysis of vocalizations in Het and Rl pups, it was possible to detect a delay in reaching the peak-day of emission (6th *vs* 4th day), associated to a delay in reaching a more adult-like vocal profile. Altogether, these findings confirm and extend the presence in reelin mutant mice of quantitative and qualitative alterations in USVs already in a neonatal phase [38], indicative of the delay on the emotional and communicative development comparable to what has been found in children with ASD [68], [69].

A number of deviations from normative motor development has been highlighted in Het and Rl mice. When tested during the first 12 days of life, mutant mice exhibited longer *curling* and longer latency to right themselves when placed on their backs than Wt littermates. These two responses are strongly associated with each other, *curling* is a vigorous side to side rolling movement while on the back and it is aimed at righting. Moreover, Rl pups showed a deficit in *face washing* and *wall climbing* at pnd 12 as compared to their Wt and Het littermates. Both these behaviors require coordination and equilibrium since pup is standing up on its hindlimb and washing its face or climbing the walls of the container with forelimbs. Altogether these data suggested the presence of a motor coordination deficit in mutant mice supported by the fact that, in the first weeks of life, mutant mice show progressive loss of Purkinje cells of the cerebellum [70], [71], a brain area that plays an important role in fine motor coordination. A genotype-induced delay in the behavioral development of Rl mice is also suggested by a decrease in *circling*, *nose probing* and *head shacking* behaviors and by the hyperactivity profile displayed by mutant pups on pnd 12. As a whole, persistence of immature motor behavioral responses characterizes the Rl pups. Such motor profile seems in line with the transient delays in sensory–motor development shown by other animal models of ASD such as Mecp2-null, Foxp2, and BTBR mice [37], [46], [47], [56], [72]. Early neurological abnormalities found in Rl and Het mice are particularly intriguing, in view of subtle alterations in infantile reflexes (such as *body righting* or *head tilting*) recently described in infants later diagnosed for autism [73]. Studies on family videos provided by parents of children later diagnosed as ASD have highlighted the presence of both subtle deficits and pre-regression developmental delays during the first months of life [74]–[76]. Infants with ASD had more often poor repertoire writhing general movements as well as abnormal or absent fidgety movements than control infants. Prospective studies performed on infant siblings of children with ASD evidenced early motor delay such as poor motor coordination, abnormal postural control and atypical movements [77]–[80].

From our results, it appears that Het and Rl mice showed delay in the vocal and motor development in line with the alterations in these two systems seen in children with ASD and considered early warning signs of ASD. It is worth to notice that in terms of gene-dosing the deficiency of reelin in Het pups led to an alteration mainly in the vocal emission while the total lack of reelin in Rl pups primarily induce alterations at motor coordination level. Motor deficits present in Rl pups at the end of the second postnatal week could be the first signs of the cerebellar alteration generally leading to death shortly after weaning.

Altogether, our results emphasize the importance of conducting behavioral phenotyping during the early developmental period in order to document the precise onset of symptoms, to identify transient signs and provide a basis for the timing of early intervention. In conclusion, our data confirm that reelin mutant mice represent an interesting animal model for behavioral abnormalities seen in neurodevelopmental disorders like autism and schizophrenia [6], [81] and highlight the importance of reelin during development.

## Supporting Information

Figure S1 *Results about pattern of sonographic structure among genotypes and sex*
**Pie graphs show the percentages of the different call categories for the three genotypes, (Wt, Het, Rl) in male pups during five days of testing (pnd 2, 4, 6, 8, 12).**
(TIF)

Figure S2 **Pie graphs show the percentages of the different call categories for the three genotypes, (Wt, Het, Rl) in female pups during five days of testing (pnd 2, 4, 6, 8, 12).**
(TIF)

Figure S3 **Production of ultrasonic vocalizations by call category. A) Frequency of ultrasonic vocalizations at pnd 4, B) pnd 8 and C) pnd 12.**
(TIF)

Materials S1 (DOCX)
