# Supplementary figures and images for: Characterization of Neonatal Vocal and Motor Repertoire of Reelin Mutant Mice
Source: PLoS One. 2013 May 21;8(5):e64407. doi: 10.1371/journal.pone.0064407 (PMC3660261; doi:10.1371/journal.pone.0064407)

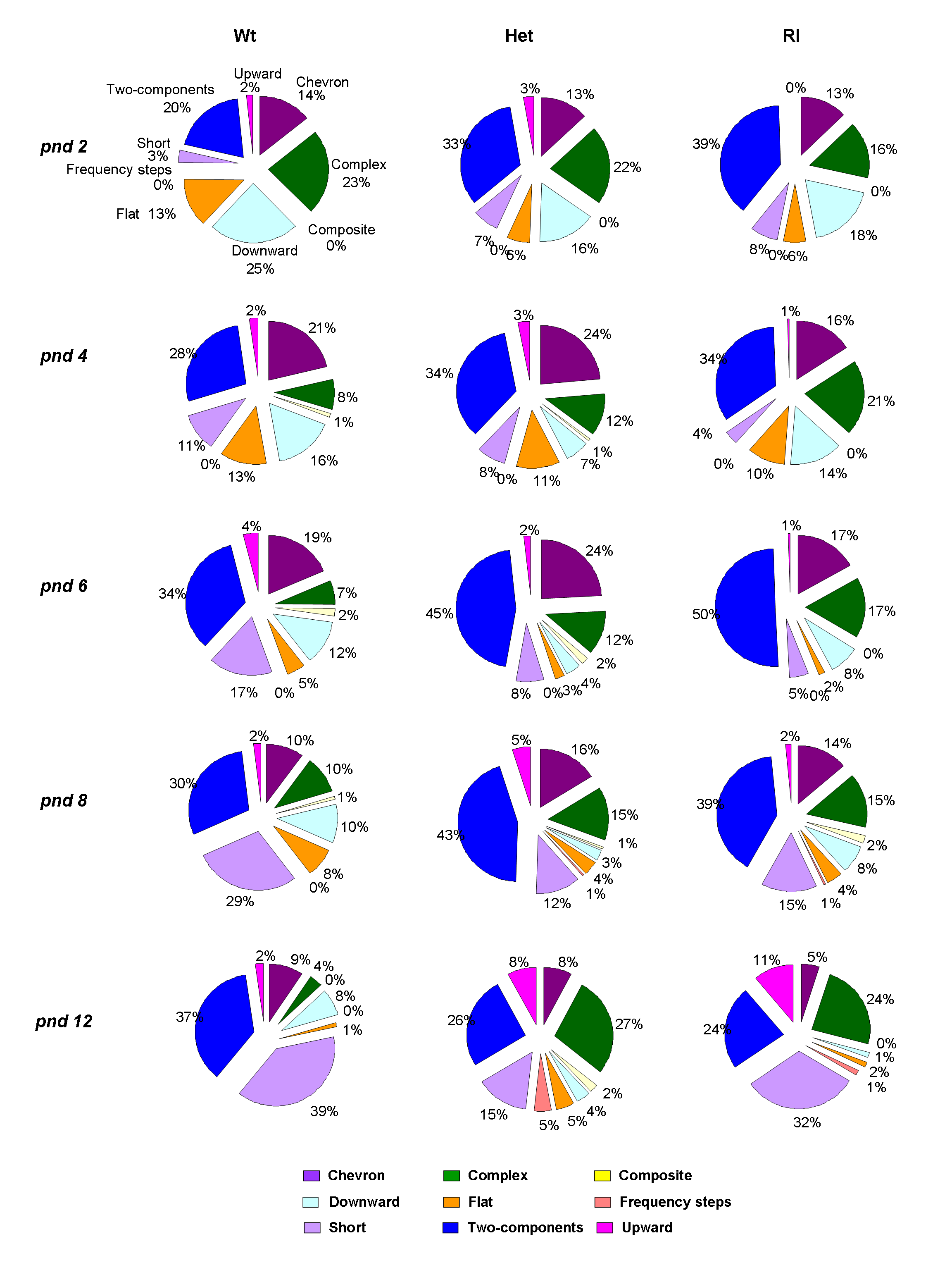

Supplement: Figure S1 — Results about pattern of sonographic structure among genotypes and sex Pie graphs show the percentages of the different call categories for the three genotypes, (Wt, Het, Rl) in male pups during five days of testing (pnd 2, 4, 6, 8, 12). (TIF) [file pone.0064407.s001.tif]

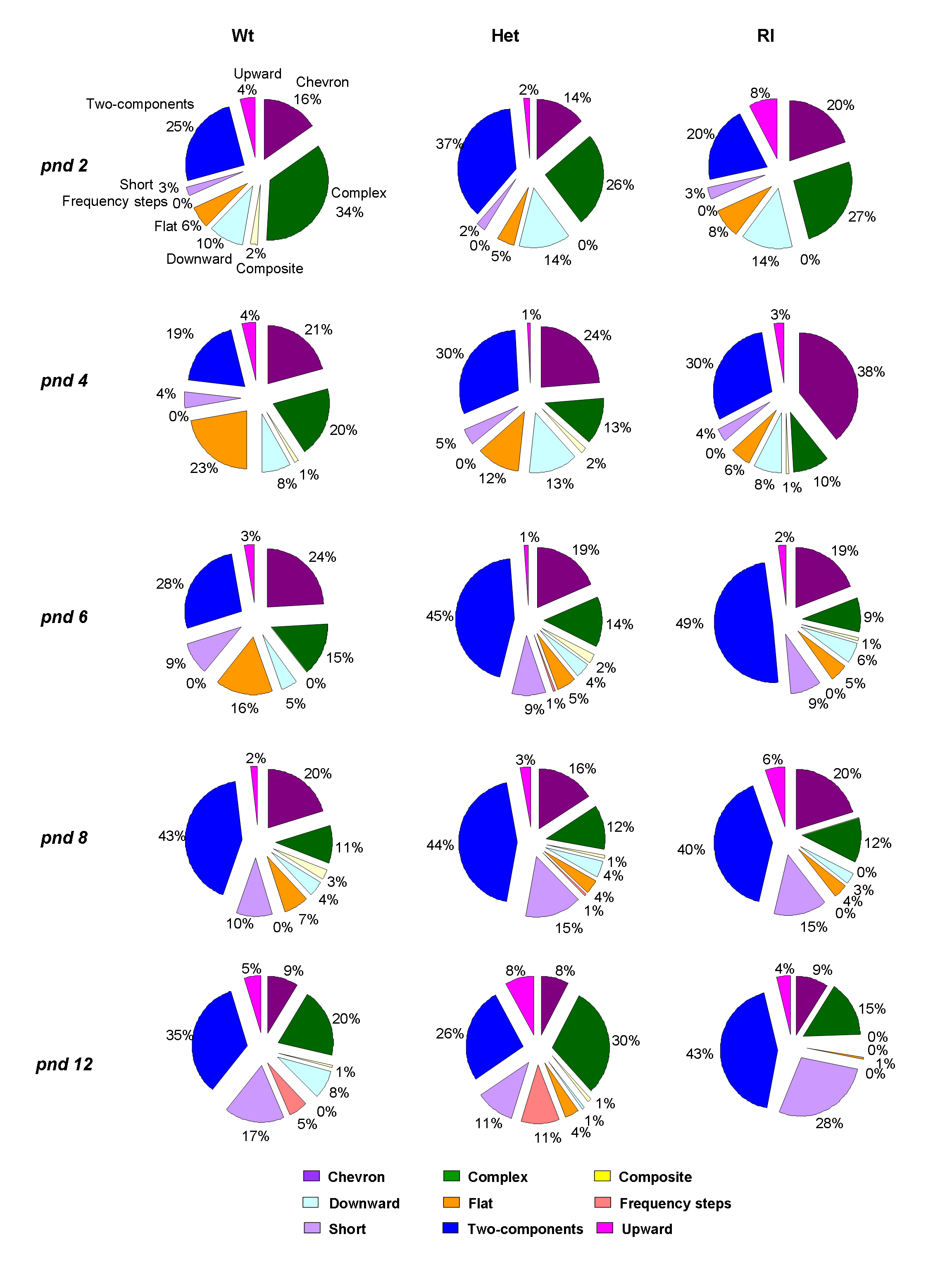

Supplement: Figure S2 — Pie graphs show the percentages of the different call categories for the three genotypes, (Wt, Het, Rl) in female pups during five days of testing (pnd 2, 4, 6, 8, 12). (TIF) [file pone.0064407.s002.tif]
