# Supplementary material for: Characterization of Neonatal Vocal and Motor Repertoire of Reelin Mutant Mice
Source: PLoS One. 2013 May 21;8(5):e64407. doi: 10.1371/journal.pone.0064407 (PMC3660261; doi:10.1371/journal.pone.0064407)
Supplement: Materials S1 — (DOCX) [file pone.0064407.s004.docx]

**Supplementary Materials**

*Pattern of sonographic structure among genotypes and sex*

Proportions of calls within each category in males are shown in Figure S1. In the first eight days of testing, Wt male pups emitted a wide range of categories (6 out of 9 categories), reducing substantially their vocal repertoire to the *short* and ***two-component*** calls at pnd 12. Het and Rl male pups (second and third column) emitted a wide range of categories until pnd 4, and limited their vocal repertoire at pnd 6 and 8 to the ***two-component*** (about 45%), *chevron* (about 20%) and *complex* calls (about 15%). On pnd 12, Het and Rl male pups still persisted in emitting primarily the ***two-component*** (about 25%) and *complex* (about 26%) calls but increased, as Wt pups, the emission of *short* calls (about 24%).

On pnd 2, Wt female pups (Figure S2) mainly emitted the ***two-component*** (25%), *chevron* (16%) and *complex* calls (34%). Later in development, a reduction in the use of *chevron* (9%) calls occurred in favor of an increased expression of *short* calls (17%)*.* As illustrated in the column of the Het and Rl females, it comes out immediately that these subjects communicate mainly through the emission of the ***two-component*** calls. Starting from pnd 8, Het's emission profile appeared comparable to that of Wt pups. While Wt and Het females emitted at pnd 12 a wide range of categories (6-7 out of 9 categories), vocal repertoire of Rl female pups at the same age was mainly characterized by the use of only 4 categories, specifically ***two-component*** (43%), *chevron* (9%)*, complex* (15%) and *short calls* (28%).
